# Seasonal Changes in Mycosporine-Like Amino Acid Production Rate with Respect to Natural Phytoplankton Species Composition

**DOI:** 10.3390/md13116740

**Published:** 2015-11-06

**Authors:** Sun-Yong Ha, Yeonjung Lee, Min-Seob Kim, K. Suresh Kumar, Kyung-Hoon Shin

**Affiliations:** 1 Division of Polar Ocean Environment, Korea Polar Research Institute (KOPRI), 26 Songdomirae-ro, Yeonsu-gu, Incheon 406-840, Korea; E-Mail: syha@kopri.re.kr; 2 Department of Marine Sciences and Convergent Technology, Hanyang University, Ansan 426-791, Korea; E-Mails: yeonjunglee83@gmail.com (Y.L.); ksuresh2779@gmail.com (K.S.K); 3 Environmental Measurement & Analysis Center, National Institute of Environmental Research, Environmental Research Complex, 42 Hwangyeong-ro, Seo-gu, Incheon 404-708, Korea; E-Mail: candyfrog77@hotmail.com

**Keywords:** chlrophyll (chl) *a*, lake, mycosporine-like amino acids (MAAs), phytoplankton, ultraviolet radiation (UVR)

## Abstract

After *in situ* incubation at the site for a year, phytoplanktons in surface water were exposed to natural light in temperate lakes (every month); thereafter, the net production rate of photoprotective compounds (mycosporine-like amino acids, MAAs) was calculated using ^13^C labeled tracer. This is the first report describing seasonal variation in the net production rate of individual MAAs in temperate lakes using a compound-specific stable isotope method. In the mid-latitude region of the Korean Peninsula, UV radiation (UVR) usually peaks from July to August. In Lake Paldang and Lake Cheongpyeong, diatoms dominated among the phytoplankton throughout the year. The relative abundance of *Cyanophyceae* (*Anabaena spiroides*) reached over 80% during July in Lake Cheongpyeong. Changes in phytoplankton abundance indicate that the phytoplankton community structure is influenced by seasonal changes in the net production rate and concentration of MAAs. Notably, particulate organic matter (POM) showed a remarkable change based on the UV intensity occurring during that period; this was because of the fact that cyanobacteria that are highly sensitive to UV irradiance dominated the community. POM cultured in Lake Paldang had the greatest shinorine (SH) production rate during October, *i.e.*, 83.83 ± 10.47 fgC·L^−1^·h^−1^. The dominance of diatoms indicated that they had a long-term response to UVR. Evaluation of POM cultured in Lake Cheongpyeong revealed that there was an increase in the net MAA production in July (when UVR reached the maximum); a substantial amount of SH, *i.e.*, 17.62 ± 18.34 fgC·L^−1^·h^−1^, was recorded during this period. Our results demonstrate that both the net production rate as well as the concentration of MAAs related to photoinduction depended on the phytoplankton community structure. In addition, seasonal changes in UVR also influenced the quantity and production of MAAs in phytoplanktons (especially *Cyanophyceae*).

## 1. Introduction

Ultraviolet radiation (UVR) reaching the Earth’s surface (280–400 nm) is known to inhibit photosynthesis and nutrient uptake [1,2,3]. Besides inhibiting protein synthesis and the activity of several enzymes, it causes DNA damage [4,5,6] in phytoplankton, which consequently results in a decrease in primary productivity of aquatic ecosystems. However, phytoplankton have developed several strategies to cope with UVR. Moreover, the magnitude of UVR mediated damage varies based on the phytoplankton species, *i.e.*, based on the photoprotective mechanisms employed by each organism [4,7].

Several organisms, including phytoplankton, produce photoprotective compounds such as mycosporine-like amino acids (MAAs) and carotenoids in order to protect their UVR-sensitive cellular components; these compounds facilitate their survival in UVR-enriched environments, for, e.g., transparent aquatic ecosystem [3,8]. MAAs are known for their UV-absorbing properties (λ_max_ = 310–362 nm), and are either biosynthesized and/or accumulated by both marine and freshwater primary producers and consumers [9,10,11,12,13]. MAAs are small (<400 Da), water-soluble compounds consisting of aminocyclohexenone with amino alcohol substituents, or aminocyclohexenimine rings [14,15].

UVR has been known to influence phytoplankton or benthic communities. Several reports on the effects of UV on living organisms in alpine lakes and their UV photobiology are available [10,12,16]; most reports suggest that the UV sensitivity varied with species or with the community structure. However, in high latitudes or high-latitude lakes (that are easily influenced by acidification and climatic warming), the effects of UV are significantly associated with environmental changes [16,17,18]. For mid-latitudinal regions, the effects of UV on aquatic organisms in the Korean Peninsula are also available [19,20,21].

This study focused on two mid-latitude freshwater lakes (Lake Paldang and Lake Cheongpyeong). Freshwater phytoplankton, collected from the surface water at these sites, was exposed to UVR, and real-time production of UV absorbing compounds (UVACs) was examined; in addition, seasonal variation in UVACs were examined. These two lakes are reported to be artificially created by the construction of dams, and have been classified as typical lakes formed by rivers [22,23]. Periodically, with varying weather and nutrient levels, since 1990, *Cyanophyceae* are reported to alternate their spread and disappearance [23]. This study aimed at ascertaining the impact of the phytoplankton community structure on the net production of UVAC (*i.e.*, MAAs) in temperate lakes. This is the first report of its kind for mid-latitude freshwater, examining *in situ* individual MAA net production rate using a compound-specific stable isotope technique.

## 2. Results

### 2.1. Seasonal Variation in Chl a and Phytoplankton Composition

In Lake Paldang, with the rise in water temperature from March until July, the chlrophyll (chl) *a* concentration increased from 17.8 to 29.8 µg·L^−1^. In July, when chl *a* concentration was at its highest level of 29.8 µg·L^−1^, the water temperature reached 27 °C. Following heavy rains in July (408 mm), the lowest chl *a* concentration (11.3 µg·L^−1^) was recorded in August. Subsequently, between September and November, the chl *a* increased from 16.7 to 24.7 µg·L^−1^ (Table 1). The highest turbidity (10.1 NTU) was recorded during August (Table 1).

**Table 1:** Seasonal variation of chlorophyll *a* concentration (μg·L^−1^), surface temperature (°C), Dissolved Oxygen (DO; mgO_2_·L^−1^), turbidity (NTU), and UV-B irradiance (mW·m^−2^) of Lakes Paldang and Cheongpyeong.

Location of Study and UV-B Intensity	Month	March	April	May	June	July	August	September	October	November
Lake Paldang	Chl *a*	25.6	17.7	17.8	19.9	29.8	11.2	16.7	24.7	18.1
	Temp.	8	15	16	23	27	23	24	18	12
	DO	12.5	14.9	10.5	10.7	11.9	9.9	11	12.6	11.8
	Turbidity	0.6	0.9	2.5	1.4	7	10.1	1.6	2.8	2.8
Lake Cheongpyeong	Chl *a*	9.6	16.9	14.4	19.9	10.4	2.6	8.5	10.5	18.7
	Temp.	8	13	14	22	26	21	23	19	11
	DO	14.7	14.1	11	10.1	10.7	9.3	10.8	8.7	12.9
	Turbidity	0.3	0.5	1.8	1.2	2.5	15.1	1.5	1.7	1.5
UV-B intensity (mW·m^−2^)		94.2	142.3	166.5	156	163.1	162.8	134.4	90.3	39.5

Chl *a* concentrations in Lake Cheongpyeong were not much different from Lake Paldang, but they generally remained lower (Table 1) except for November. In case of Lake Cheongpyeong, in July, when the maximum water temperature was recorded, the chl *a* concentration reached 10.4 µg·L^−1^. During August (in the presence of heavy rains), the lowest chl *a* (2.6 µg·L^−1^) and highest turbidity (15.1 NTU) were witnessed (Table 1). Between March and July, the chl *a* concentration increased from 9.6 to 19.9 µg·L^−1^, which was lower than Lake Paldang. The highest chl *a* concentrations were observed in June. From August, the chl *a* concentrations kept increasing, ranging from 8.5 to 18.7 µg·L^−1^.

In Lake Paldang, diatoms were the dominant species during March. However, in April, other flagellates began to appear and had a relative abundance of over 30%. During the same period of time, *Cyanophyceae* and *Chlorophyta* appeared but their numbers were infinitesimal. Diatoms and other flagellates increased in abundance during May; however, this increase was temporary as the proportion of *Cyanophyceae* (*Pseudoanabaena* spp.) exceeded 30%. Until mid-June, the total population of phytoplankton was <6000 cells/mL; at this time, the diatoms were relatively abundant. Because *Cyanophyceae* and *Chlorophyta* populations began to increase dramatically in July (when the water temperature was approximately 20 °C), ecological succession occurred in phytoplankton. *Cyanophyceae* species observed during this time were *Anabaena* spp. and *Microcystis* spp., both of which are known to cause water bloom and produce toxins (Table 2).

**Table 2:** Relative composition (%) of phytoplankton community of Lake Paldang.

Data Species	March	April	May	June	July	August	September	October	November
**Bacillariophyceae**									
*Achanthoseras zachariasii*								1.06	
*Achnanthes* sp.	0.16								
*Amphora ovalis*									0.11
*Amphora* sp.									
*Asterionella formosa*	2.28	24.96	53.90	20.18	0.10			2.12	2.35
*Aulacoseira granulata*					2.57				
*Aulacoseria granulata* var. *angustissima*	0.90			47.96	36.56	12.61	41.27	66.03	64.14
*Cocconeis placentula*									0.11
*Cocconeis* sp.	0.08								
*Cyclotella comta*	1.06					2.50			
*Cyclotella meneghiniana*			0.12	2.80	4.53	17.50	0.45	2.55	2.13
*Cyclotella pseudostelligra*		2.60							8.75
*Cyclotella stelligera*			0.12	9.77					
*Cyclotella* sp.					1.51	5.00	4.99	6.79	7.58
*Cymbella affinis*	0.24								
*Cmbella minuta* var. *silesiaca*		0.35						0.42	
*Cymbella tumida*	0.49								
*Cymbella turgidula*						0.43			
*Fragilaria crotonensis*		5.03	24.15						1.49
*Fragilaria* sp.									0.43
*Gomphonema* sp.									
*Hantzschia* sp.						0.21			
*Navicula cryptocephala*	0.20							0.85	
*Navicula gregaria*	0.05					1.28			0.21
*Navicula pupula*						1.28			0.21
*Navicula viridula* var. *rostellata*								0.21	
*Navicula* sp.									0.11
*Nitzschia acicularis*	1.22	4.94	0.30	1.06	0.23	4.49			0.21
*Nitzschia actinastroides*				1.59	0.19				
*Nitzschia amphibia*		1.65				2.24			0.32
*Nitzschia palea*	1.22			1.06		6.73	0.79	1.06	0.32
*Nitzschia sinuata* var. *tabellaria*			0.30						
*Nitzschia* sp.	0.41							0.21	0.21
*Rhizosolenia eriensis* var. *morsa*								0.21	
*Stephanodiscus hantzschii*	89.50	57.02	3.27	6.19	0.83				0.11
*Synedra acus*			0.12	4.60	0.21	0.43	1.47	0.21	
*Unknown*			0.35					2.76	
subtotal	97.80	96.53	82.62	95.22	46.72	54.70	48.98	84.50	88.79
***Cyanophyceae***									
*Anabaena macrospora*					17.13				
*Anabaena spiroides*					3.07				
*Anabaena smithii*							8.16		
*Anabaena sp.*				4.25	3.84				
*Aphanizomenon flos-aquae*					4.68			3.82	
*Merismopedia grauca*						13.68	35.15		
*Microcystis wesenbergii*					3.12				
*Microcystis* sp.								4.67	
*Pseudoanabaena* sp.						13.68			1.92
*unknown*						0.21			
subtotal				4.25	31.84	27.56	43.31	8.49	1.92
**Chlorophyceae**									
*Ankistrodesmus falcatus* var. *mirabilis*	0.33	0.52	0.12	0.53	4.89	0.43	0.45		0.64
*Characium* sp.					0.83				
*Chlamydomonas umbonata*									0.11
*Chlamydomonas* sp.			0.82				0.23		
*Chlorogonium elongatum*							0.11		
*Chlorogonium* sp.									
*Coelastrum* sp.			1.87			1.71			
*Cosmarium* sp.							0.11		
*Dictyosphaerium ehrebergianum*						3.42			
*Eudorina elegans*					1.66				
*Hormidium* sp.									5.12
*Lobomonas rostrata*					0.10				
*Micractinium pusillum*	0.49					0.85	0.34		
*Oocystis* sp.			0.35						
*Pandorina morum*					8.12		0.91		
*Pediastrumsimplex* var. *simplex*							0.91		
*Pteromonas* sp.							0.45		
*Scenedesmus acuminatus*					1.42				
*Scenedesmus acutus* f. *costulatus*		2.77				3.59			0.43
*Scenedesmus ecornis*						3.59			0.85
*Scenedesmus quadricauda*					2.12	1.79	2.04		
*Scenedesmus* sp.	0.98								
*Staurastrum* sp.							0.11		
*Treubaria schmidlei*							0.45		
Unknown			0.12			0.43			
subtotal	1.79	3.29	3.27	0.53	19.15	15.81	6.12		7.15
**Others**									
*Cryptomonas ovata*	0.16		7.91						1.28
*Cryptomonas* sp.			1.31		0.21	1.71		0.85	0.21
*Komma* sp.			0.23						
*Mallomonas raginae*					0.05				
*Mallomonas* sp.	0.24		0.23		0.05		0.11		0.11
*Peridinium bipes* f. *occultatum*			0.58		1.98			4.67	0.21
*Peridinium* sp.		0.17					0.11	1.49	
*Phaccus* sp.									
*Rhodomonas* sp.			3.85			0.21	1.36		0.32
subtotal	0.41	0.17	14.12		2.29	1.92	1.59	7.01	2.13

In Lake Cheongpyeong, both the highest populations of phytoplankton, and, the highest levels of chl *a* concentrations, occurred during June. Diatoms comprised the highest percentage of phytoplankton every month, except July (Table 3). *Chlorophyta*, other algae and *Cyanophyceae* appeared during the rainy season in summer, but their number was infinitesimal. In this lake, diatoms were dominant; they made up at least 80% of the phytoplankton composition by June. In July, the *Cyanophyceae* succeeded the diatoms, showing a similar value.

**Table 3:** Relative composition (%) of phytoplankton community of Lake Cheongpyeong.

Data Species	March	April	May	June	July	August	September	October	November
**Bacillariophyceae**									
*Achanthoseras zachariasii*							6.69	2.49	0.62
*Achnanthes* sp.						0.97		0.17	
*Amphora* sp.						0.97			
*Asterionella formosa*	8.39	8.39	59.38	46.19		7.77	0.65	21.33	0.42
*Aulacoseira granulata*							4.84	2.11	
*Aulacoseria granulata* var. *angustissima*				1.24	4.71	10.68	12.34	45.43	13.75
*Cyclotella comta*			0.57				0.24		
*Cyclotella meneghiniana*			0.19	0.21				2.77	0.62
*Cyclotella pseudostelligra*									46.24
*Cyclotella stelligera*	0.71	0.71		0.65		11.17	21.21		
*Cyclotella* sp.						8.74		4.88	
*Cymbella affinis*						2.43			
*Cymbella minuta* var. *silesiaca*	0.35	0.35							
*Cymbella turgidula*							0.24		
*Cymbella* sp.			0.09			1.46			
*Fragilaria construens* f. *venter*	1.74	1.74				4.37			
*Fragilaria crotonensis*	6.06	6.06	22.16	37.81	2.02	5.83			8.12
*Fragilaria* sp.									0.21
*Melosira varians*						0.97			
*Navicula cryptocephala*						0.49			
*Navicula gregaria*	0.12	0.12				1.94			
*Navicula viridula* var. *rostellata*									
*Navicula* sp.	0.12	0.12	0.19			5.83	0.24	0.83	
*Nitzschia acicularis*	2.01	2.01	1.70		0.47	3.88	2.42	0.28	1.87
*Nitzschia levidensis* var. *levidensis*						0.49			
*Nitzschia palea*					1.41	16.99	1.05		
*Nitzschia sinuata* var. *tabellaria*						0.49			
*Nitzschia* sp.			0.57	0.38		3.88			
*Rhizosolenia eriensis* var. *morsa*			0.28				4.27	0.83	0.02
*Stephanodiscus hantzschii*	67.85	67.85	9.47						
*Synedra acus*			0.57					1.55	
*Synedra ulna*						0.97			
*Unknown*						0.97			
subtotal	87.35	87.35	95.17	86.48	8.61	91.26	54.19	82.66	71.88
***Cyanophyceae***									
*Anabaena spiroides*					40.09				
*Anabaena sp.*				0.29	17.79		0.65	0.83	
*Microcystis aeuriginosa*					16.80				
*Microcystis wesenbergii*								9.14	
*Pseudoanabaena* sp.					7.39				
subtotal				0.29	82.07		0.65	9.97	
**Chlorophyceae**									
*Ankistrodesmus falcatus* var. *mirabilis*			0.09			0.97	3.06		0.62
*Carteria cordifomis*							1.21		
*Characium* sp.				4.10					
*Chlamydomonas umbonata*					0.61				0.21
*Chlamydomonas* sp.	0.35	0.35	0.09				0.65		
*Closteriopsis longissima*							0.24		
*Dictyosphaerium ehrebergianum*							2.42		
*Elakatothrix gelatinosa*					0.14				
*Eudorina elegans*					2.49				
*Hormidium* sp.						2.91	7.26		21.87
*Micractinium pusillum*							0.81		
*Pediastrum duplex* var. *reticulatum*								2.22	
*Pediastrum* sp.					1.27				
*Scenedesmus acutus* f. *costulatus*								0.55	
*Scenedesmus ecornis*					0.61				
*Scenedesmus denticulatus*									0.83
*Scenedesmus quadricauda*							0.81	2.22	0.83
*Scenedesmus spinosus*					0.61				
*Scenedesmus* sp.								0.28	
*Staurastrum* sp.							4.44		
*Treubaria* sp.	0.12	0.12							
Unknown				1.05	0.14				0.62
subtotal	0.47	0.47	0.19	5.14	5.88	3.88	20.89	5.26	24.99
**Others**									
*Cryptomonas ovata*	0.71	0.71	3.98	1.05	1.18		6.29	0.28	
*Cryptomonas* sp.					1.18	0.97			0.83
*Komma* sp.									0.83
*Mallomonas raginae*				0.10					
*Mallomonas* sp.	0.35	0.35	0.09				0.81		
*Peridinium bipes* f. *occultatum*			0.57	6.19	1.08				1.04
*Peridinium* sp.						1.46	6.05	1.11	0.42
*Rhodomonas* sp.	11.11	11.11		0.76		2.43	11.13	0.72	
subtotal	12.17	12.17	4.64	8.10	3.44	4.85	24.27	2.11	3.12

### 2.2. Seasonal Variation in Concentration and Net Production Rate of MAA

**Figure 1:**
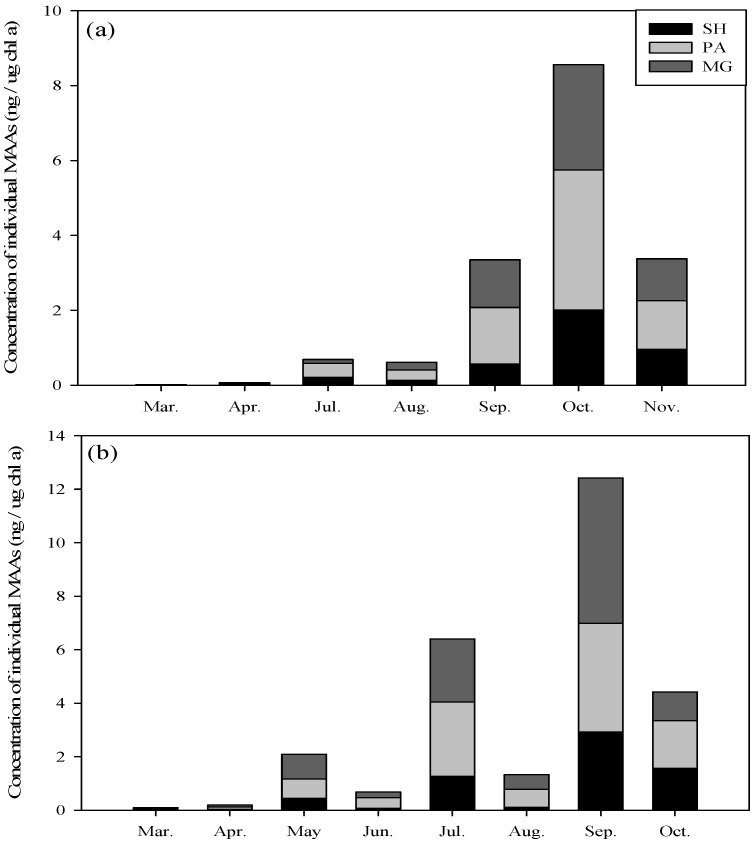
Seasonal change in natural concentration of individual MAAs in Lake Paldang (**a**) and Cheongpyeong (**b**). SH: Shinorine (black); PA: Palythine (gray); MG: Mycosporine-glycine (dark gray).

In Lake Paldang, relatively high MAA concentrations, *i.e.*, 8.56 ± 0.09 ng/μg chl *a*, were observed in October. In September and November, MAA concentrations of 3.35 ± 0.21 ng/μg chl *a* and 3.38 ± 0.12 ng/μg chl *a*, were respectively observed. On the other hand, the MAA concentration was 0.02 ± 0.002 ng/μg chl *a* to 0.61 ± 0.17 ng/μg chl *a* from March to August. During May, no MAA concentration was detected on some occasions. Amongst the MAA, the MG and PA were high every month, while the SH were detected at relatively low concentrations (Figure 1).

In Lake Cheongpyeong, the highest MAA concentration, *i.e.*, 12.42 ± 0.98 ng/μg chl *a*, was detected in September. In July and October, the MAA concentrations were 6.4 ± 0.96 ng/μg chl *a* and 4.42 ± 0.24 ng/μg chl *a*, respectively. On the other hand, from March to June, the MAA concentration ranged from 0.09 ± 0.01 to 0.68 ± 0.09 ng/μg chl *a*. In August, the MAA concentration was <1.33 ± 0.0.59 ng/μg chl *a* (Figure 1). In general, the MAA concentration in Lake Cheongpyeong was higher than Lake Paldang, which indicated that the two lakes differ in their phytoplankton community structure.

**Figure 2:**
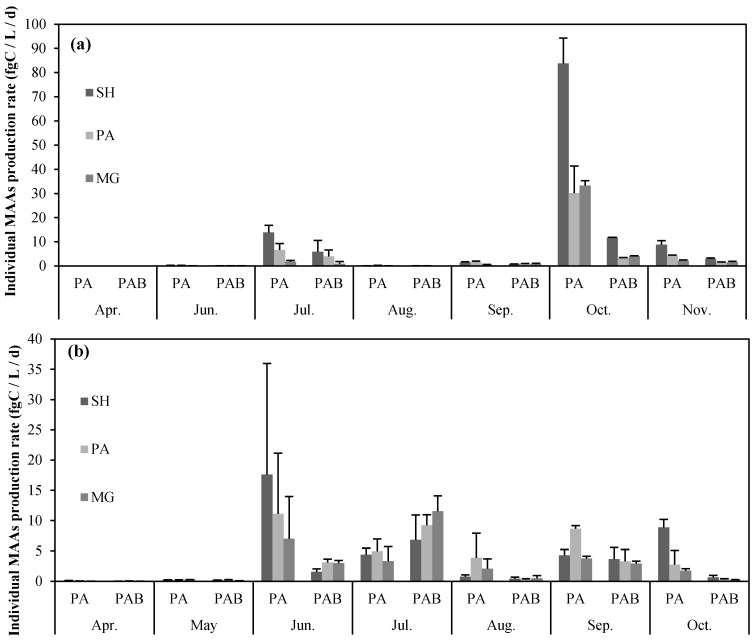
Individual MAAs production rate in (**a**) Lake Paldang and (**b**) Lake Cheongpyeong. PA: exposed to PAR+UV-A; PAB: exposed to PAR+UV-A+UV-B; SH: Shinorine (black bars); PA: Palythine (gray bars); MG: Mycosporine-glycine (light gray bars).

Irrespective of exposure to UVR, the highest MAA net production rates were observed from POM in Lake Paldang during October. Notably, the POM exhibited maximum net production rate even when UVR was cut off. Here, the concentrations of SH, PA and MG were 83.83 ± 10.47, 30.18 ± 11.2 and 33.26 ± 2.01 fgC·L^−1^·h^−1^, respectively. Particularly, the POM exposed to UVR during October demonstrated SH, PA and MG of 11.82 ± 0.01, 3.19 ± 0.3 and 4.02 ± 0.22 fgC·L^−1^·h^−1^, respectively. Based on these observations, it could be stated that the net production rates of UV-exposed phytoplankton were higher than those of the phytoplankton not exposed to UV (Figure 2). During all months, except October, the POM exhibited very low productivity; moreover, in July and November, slightly higher productivity rates were observed for the POM.

In Lake Cheongpyeong, during June and July, relatively high MAA net production rates were recorded. In particular, the highest MAA net production rate was observed in the POM that was not exposed to UVR during June; especially at this time, highest SH production was exhibited, *i.e.*, 17.62 ± 18.34 fgC·L^−1^·h^−1^ (Figure 2). In June, when the UVR was maximum, the MAA net production rate was higher in POM exposed to UVR, rather than the unexposed samples. Moreover, amongst the MAA, high MG production rates (*i.e.*, 11.55 ± 2.53 fgC·L^−1^·h^−1^) were observed, rather than SH. During July, in the case of the POM that was not exposed to UVR, the SH, PA and MG net production rates did not differ much; they were 4.39 ± 1.07, 4.94 ± 2.03 and 3.32 ± 2.39 fgC·L^−1^·h^−1^, respectively. The lowest net production rate of MAAs was observed from April to May. During September and October, the MAA net production rate was relatively low (Figure 2).

## 3. Materials and Methods

### 3.1. Study Area

**Figure 3:**
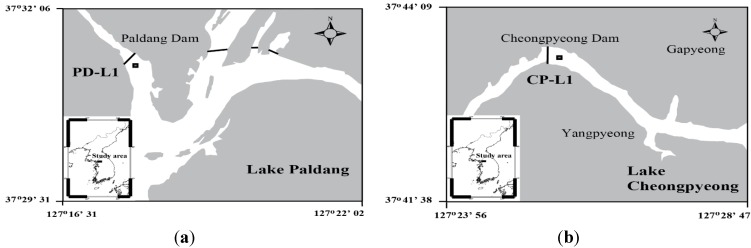
Location of the study area: (**a**) Lake Paldang and (**b**) Lake Cheongpyeong.

Lake Paldang is a man-made lake located in the central part of the Korean peninsula (37°35′48″ N, 12°21′3″ E) created by a hydroelectric dam; the North Han River, the South Han River and the Kyungan River meet at this location. Its lake basin measures 23,800 square kilometers and the total reservoir capacity reaches 2.44 million tons. Water mass has a short residence time, and the depth of the water averages 6.55 m, causing poorly developed stratification (Figure 3a). On the other hand, Lake Cheongpyeong, formed by another dam, comprises the lower reaches of the North Han River (37°47′50″ N, 127°31′10″ E). The mainstream of the North Han River, the Hongchon River and the Gapyong River connect at Lake Cheongpyeong (Figure 3b). Its lake basin measures 9.921 square kilometers, while its water surface area reaches 17.6 square kilometers. As the water flows in only when the floodgates are opened in both these lakes (Paldang and Cheongpyeong), they do not function as reservoirs; therefore, they have a fairly constant water level all year.

Based on the data provided by the Climate Change Information Center (http://www.climate.go.kr/home/Eng/htmls/kgawc/sub1.html) and the Gangneung Meteorological Administration (Table 1), a seasonal variation in the UV-B intensity was observed during the study period. The average UV-B intensity recorded during 2008 was 114.7 mW·m^−2^. Low UV-B intensity was recorded during the winter months, while high intensities were observed in July (196 mW·m^−2^) and August (191.3 mW·m^−2^). The Han River Environment Research Center carried out various analyses of the water samples of the two lakes *viz*. phytoplankton identification, quantitative analysis of phytoplankton, estimation of chlorophyll (chl) *a* concentration and physiochemical analyses of water (YSI-6600, YSI Inc., Yellow Springs, OH, USA), especially turbidity (NTU) analysis.

### 3.2. In Situ Culture Experiment Using ^13^C Tracer

**Figure 4:**
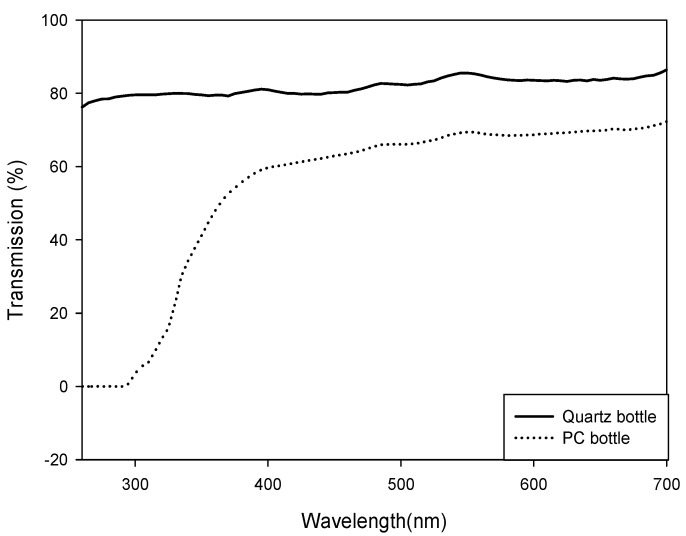
Transmission of UV-B radiation by Polycarbonate and Quartz bottles.

In order to determine the MAA concentration and net production rate during natural UVR exposure, *in situ* incubation experiments were conducted at sites in Lake Paldang and Lake Cheongpyeong (Figure 3). Surface water was collected from March to November 2008 on a monthly basis at the sampling stations of Lake Paldang and Lake Cheongpyeong; the water samples were transferred to quartz (HanJin Quartz Co., Seoul, South Korea) and polycarbonate bottles (PC; Nalgene^®^Labware, Rochester, NY, USA) in duplicate for *in situ* incubation analysis. Quartz bottles were used for PAR+UV-A+UV-B (PAB) radiation treatment while PC bottles (which block the transmission of UV-B radiation) were used for PAR+UV-A (PA) radiation treatment (control). The transmission spectra of quartz and PC bottles (Figure 4) were measured by spectrophotometer (Cary^®^ 50, Agilent, Santa Clara, CA, USA). An *in situ* culture experiment using ^13^C tracer was performed according to Hama [24]. (^13^C)-Labeled sodium bicarbonate, *i.e.*, NaH^13^CO_3_ (99%), which was added as a tracer, increased up to 15% of the ^13^C in the total dissolved inorganic carbon pool. To observe the effects of UVR, the incubation bottles were then immersed at the surface (within 1 m) of the seawater at the *in situ* sampling stations of Lake Paldang and Lake Cheongpyeong; here, the bottles remained incubated for 4 h at mid-daylight (from 10 am to 2 pm). After exposure, incubated samples were filtered using pre-burned (450 °C, 4 h) GF/F 47-mm filter paper. They were then transported to the laboratory using a liquid nitrogen container; the filtered samples were stored at −80 °C until they were analyzed.

### 3.3. Extraction and Analysis of Mycosporine-Like Amino Acids

**Figure 5:**
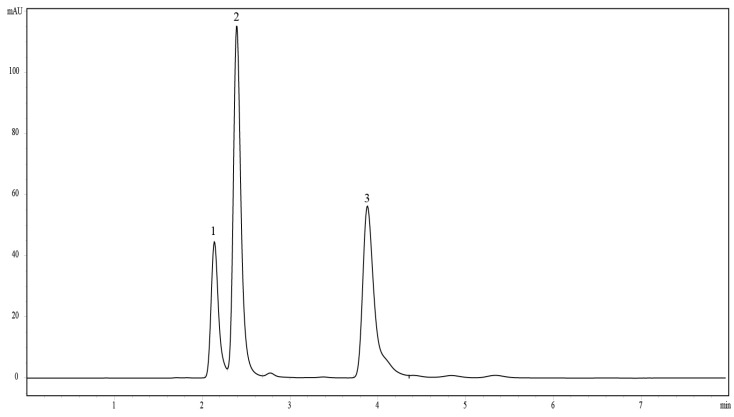
A typical HPLC-DAD chromatogram and absorption spectrum of individual MAAs in the present study; 1: shinorine (λ_max_ = 334); 2: palythine (λ_max_ = 320); 3: mycosporine-glycine (λ_max_ = 310).

MAAs in the POM were analysed by lyophilizing (lyophilizer; FD Series, IlshinBioBase, Gyeonggi-do, South Korea) the filtered samples; thereafter, 3 mL of 100% methanol (MeOH) was added to the dried samples. These samples were homogenized using an ultrasonicator (30 s, 50 W; Ulsso Hi-tech ULH-700s, Chongwon-gun, Chungcheongbuk-Do, South Korea), after which they were kept at 4 °C (overnight means 12 h;) for the extraction of MAAs. These samples were then filtered using a syringe filter (PTFE 0.20 μm Hydrophobic, Darmstadt, Germany) into a 2 mL microtube. The supernatants were evaporated to dryness at 45 °C in a centrifugal evaporator (EYELA, CVE-200D, Tokyo, Japan) and the extracts were re-dissolved in distilled water (500 μL). Thereafter, chloroform (100 μL) was added to this solution, followed by centrifugation (10,000 rpm, 10 min); then, 400 μL of the aqueous phase was carefully transferred to a new Eppendorf tube. Further analysis of the extracted MAAs was carried out using a High-Performance Liquid Chromatography (HPLC) system (Agilent Technologies 1200 series; column: Waters 120DS-AP (5 μm) 150 mm × 4.6 ID; Santa Clara, CA, USA). The detector was an Agilent DAD (G1315D; Santa Clara, CA, USA) at 313 nm (250–750 nm scan). The separated MAAs (e.g., shinroine (SH), palythine (PA), and mycosporine-glycine (MG)) were collected using a fraction collector (Agilent analyte (G1364C) FC). The samples (100 μL) were injected into the HPLC column by an autosampler (Agilent). The mobile phase, comprising 0.1% acetic acid in double distilled water, was used at a constant flow rate of 0.8 mL·min^−1^. Shinorine and porphyra-334 were used as standard references for identification and quantification of MAAs (Figure 5).

### 3.4. Calculation of the Net Production Rate and Turnover Rates of MAAs

The net production rates of individual MAAs were calculated using a modified version of the equation from Hama *et al.* [24], and the chl *a* specific production rate of each MAA was obtained by normalizing with chl *a* concentration. To determine the net production rate of each MAA, the MAA compounds were collected through a tin cap (including the piece of pre-burned filter paper). Once the solvents were completely removed by a centrifugal evaporator (EYELA, CVE-200D), the ^13^C value of each compound was measured using EA-IRMS (EuroEA-Isoprime IRMS, GV Instruments, Cheadle Hulme Stockport, UK). The net production rate of each MAA was calculated from each ^13^C atomic percent and concentration using the modified equation proposed by Hama *et al.* [24] and Ha *et al.* [25].

(1) $$\Delta \text{MAC }\left(\text{t}\right)=\text{MAC}\times \frac{a_{is}-a_{ns}}{a_{ic}-a_{ns}}$$

where ΔMAC: The amount of each MAA carbon photosynthetically produced during the incubation; *a_is_*: ^13^C atom % in each MAA compound of incubated sample; *a_ns_*: ^13^C atom % in each MAA compound of the natural sample; *a_ic_*: ^13^C atom % in the ^13^C enriched inorganic carbon; MAC: Concentration of each MAA carbon at the end of incubation.

The possible isotopic discrimination against ^13^C during photosynthetic uptake was not considered in this study because its correction did not have a significant effect on the uptake rate [24].

### 3.5. Statistics

All measurements were made in triplicates (from different cultures), and the results were expressed as mean and standard errors. Data from each individual collection time were analyzed using *t-*test, and *p* values of <0.05 and <0.01 were considered to denote statistically significant differences.

## 4. Discussion

Diatoms dominated the phytoplankton community structure throughout the year (Table 2). *Stephanodicus hantzschii* comprised 57% to 85.9% of the community from March to April, and *Aulacoseira granulate* var. *angustissima* species comprised 29.9% to 66% of the community from June to November. However, during August, *Cyclotellameneghiniana* was the dominant species. In mid-June, *Cyanophyceae* began to increase dramatically in number, leading to changes in the phytoplankton community structure in Lake Paldang (Table 2).

In the Korean Peninsula, the UVACs are more actively produced in phytoplankton from July to August, *i.e.*, when the UV-B radiation is intense. In August, heavy rains (408 mm) and the inflow of turbid water into the water system (Table 1) hinder phytoplankton growth. Generally, the inflow of turbid water lowers UV intensity and weakens the effect of UVR. Additionally, inflow of dissolved organic matter (DOM) from land also reduces UV transmission [3].

*Cyanophyceae*, containing abundant UVACs, especially *Anabaena* spp. and *Microcystis* spp., began to appear in June [26,27,28]. The cyanobacterium *Microcystis aeruginosa* is known to cause water blooms in the mid- and lower-latitudinal regions; the MAA concentration of *Microcystis aeruginosa* is reported to be higher than marine dinoflagellates (a bloom-forming phytoplankton species) [26,29]. *Anabaena* spp. are reported to produce MAAs as a protective mechanism when exposed to UVR; these MAAs function as an antioxidant [27,30,31].

Among the diatoms, *Aulacoseira granulate* var. *angustissima* dominated from June to November; its relative abundance in June, July and October, was 29.9%, 36.6% and 66%, respectively. On exposure to increasing UV intensity, the net production of UVACs in *Aulacoseira granulate* var. *angustissima* is more likely to be stimulated with time. With regard to MAA production, Zudaire and Roy [32] reported that the diatom *Thalassiosira weissflogii* had a long-term response, and showed the highest production rate after 29 days of exposure to strong UV intensities. Diatoms have long-term response to UVR as the precursors of MAAs are created via the shikimic acid pathway in this case [33]. The phytoplankton exposed to UVR prioritizes repair of its photosynthetic machinery and produces MAAs [29]; reports suggest that as this photosynthetic mechanism is repaired, there is a possible increase in MAA production [34]. In this study, changes in the external environment (UVR) were coupled with time-based changes in phytoplankton MAA production (Figure 4). Another reason for the phytoplankton to have a long-term response was that the MAAs are produced behind photoprotective xanthophyll pigments; this protects the cells against photoinhibition [32,35,36]. In phytoplankton exposed to strong light or stress, the photoprotective pigments are activated first; the MAA protects the matrix against strong light [32]. Several studies demonstrate an interactive photo-protection mechanism occurring between xanthophyll pigments and MAAs [35,36,37]. Korbee *et al.* [35] reported that in the case of the phytoplankton *Heterocapsa* sp. (containing a lot of N-substrate) photoprotective pigments were hardly produced; however, N-substrate-based MAAs conspicuously increased when the phytoplankton was exposed to strong UVR. The relationship between MAAs and nitrogen availability are reported to have either positive or negative effects on macroalgae [38,39] and microalgae [35]. The MAAs accumulation is affected by nitrogen supply as a nitrogen reservoir [40,41]. Ha *et al.* [36] reported that UVACs and the photoprotective pigment diadinoxanthin showed different reactions with time; moreover, the photoprotective pigments responded more quickly to harmful light compared to MAAs.

In this study, the net MAA production rate was higher in the POM exposed to photosynthetically active radiation (PAR), rather than the POM exposed to natural UVR. As compared to UV-A- and UV-B-exposed diatoms, the diatoms exposed to PAR demonstrate high concentrations of UVACs [14,36]. Meanwhile, in the case of the dinoflagellate *Gyrodinium dorsum*, the *in vivo* accumulation of MAAs has been reported to be actively induced by PAR or UV-B radiation [33,42]. Karsten *et al.* [43] also reported that MAAs (such as SH), were specifically induced by exposure of samples to UV-A radiation. This could be explained as follows. The intracellular composition of MAAs varies depending upon UV-B [44] and UV-A [45] radiation; moreover, each MAA compound is influenced by the wavelength of light, and each phytoplankton has different photoreactions to the MAA-synthesized mechanism [14]. In our study on diatoms, the ^13^C tracer revealed that the MAAs were more actively induced under exposure to PAR rather than PAB (Figure 4).

The phytoplankton community structure of Lake Cheongpyeong differed greatly from Lake Paldang (Table 3). In Lake Cheongpyeong, *Stephanodiscus hantzscii* dominated between March and April; but between May and June, *Asterionella formas* was dominant. This shows the succession of diatoms. In July, *Anabaena spiroides*, a harmful *Cyanophyceae*, made up the highest percentage of phytoplankton with 40.1%, but in the beginning of August (when the heavy rains prevailed), diatoms began to dominate. Diatoms continued to be dominant until December. The highest MAA concentration was observed in September, wherein high MG was recorded. Unlike other months, the chlorophyceae contributed greatly to the phytoplankton community structure during September. The relative abundance of diatoms reached 54.1%, but the abundance of chlorophyceae was 20.9%. Other phytoplankton species, *i.e.*, excluding the species analyzed, showed the highest abundance ratio during September (24.3%). Certain researchers report that MAAs in chloropyhta were actively induced by UVR [9]. MAAs and photoprotective pigments are actively produced by freshwater phytoplankton communities in many freshwater lakes [11]. Phytoplankton showed the highest MAA concentration in September; however, the MAAs that were labeled in real-time by the ^13^C tracer after exposure to natural UV-B radiation indicated the highest net production rate of MAAs between June and July (Figure 4). In July, the relative abundance of *Anabaena spiroides*, a species of *Cyanophyceae*, reached 82%, which clearly showed the correlation between the MAA net production rate and UV exposure. *Anabaena* sp., a species of *Cyanophyceae*, is known to contain SH, which is induced by UV-B radiation [13]. MAAs protect the internal organelles and cellular components against harmful UVR [4,13]. The fact that MAA production in *Anabaena* sp. increased in July (when UVR is high in the Korean Peninsula) is direct evidence that MAA functions as a photoprotector (Figure 4). However, during the other months, the MAA production of the PAR exposed POM sample was more than the UV-B exposed POM. PAR stimuled MAA net production; moreover, the dominant diatoms exercised great influence on the net production of MAAs. It is also essential to consider the fact that the MAA net production rates vary with phytoplankton species [14].

This investigation reveals seasonal variation in phytoplankton community structure and UVACs; it also demonstrated varying MAA net production rates in phytoplankton exposed to natural UVR. Seasonal variation in the MAA concentration closely influenced the phytoplankton community structure. Other reports also suggest that MAA synthesis and photoinduction are greatly influenced by seasonal changes in UVR intensity and interspecies differences in phytoplankton [14]. In our study, the *Anabaena* sp. (a bloom-forming *Cyanophyceae*), being hypersensitive to UVR, produced MAA on exposure to UVR. *Cyanophyceae*, which fixes nitrogen, produces N-substrate-based MAAs more easily than other phytoplankton species. In spite of exposure to strong UVR, diatoms in Lake Paldang had a long-term response, which is quite unexpected as *Cyanophyceae* generally have a short-term response. It is evident that the MAA of diatoms function as photo-protectors by interacting with photo-protective pigments. A future investigation involving an on-site real-time comparison between the net UVAC and photo-protective pigment production rate would help explain the selective photo-protective strategies of phytoplankton in the fresh-water ecosystem.
